# Prevention and management of diarrhea associated with naldemedine among patients receiving opioids: a retrospective cohort study

**DOI:** 10.1186/s12876-020-1173-z

**Published:** 2020-01-31

**Authors:** Yusuke Takagi, Gakuji Osawa, Yoriko Kato, Eri Ikezawa, Chika Kobayashi, Etsuko Aruga

**Affiliations:** 1 Department of Palliative Medicine, Teikyo University School of Medicine, 2-11-1 Kaga, Itabashi-ku, Tokyo, 173-8605 Japan; 2 Toda-chuo General Hospital, 1-19-3 Honcho, Toda City, Saitama, 335-0023 Japan; 3 Saiseikai Kawaguchi General Hospital, 5-11-5 Nishi-kawaguchi, Kawaguchi City, Saitama, 332-8558 Japan

**Keywords:** Adverse events, Diarrhea, Naldemedine, Opioid-induced constipation, Peripherally-acting mu-opioid receptor antagonist

## Abstract

**Background:**

Naldemedine, a novel peripherally-acting mu-opioid receptor antagonist, has improved opioid-induced constipation in randomized controlled trials. The most frequent adverse event of naldemedine is diarrhea, which can cause abdominal pain and often leads to treatment discontinuation. We aimed to identify risk factors and appropriate management strategies for key adverse events including diarrhea associated with naldemedine, since those have not been extensively studied.

**Methods:**

We conducted a multi-center retrospective cohort study. Eligible patients had cancer, had undergone palliative care at participating centers, had been prescribed regular opioids, and had taken at least one dose of naldemedine between June 2017 and March 2018. The primary endpoint was the incidence of diarrhea according to baseline characteristics. Secondary endpoints included the duration of naldemedine administration, daily defecation counts before and after starting naldemedine, duration and severity of diarrhea as an adverse event of naldemedine, other adverse events, and the incidence of constipation within 7 days after recovery from diarrhea. We defined patients who started naldemedine within three days of starting a regularly prescribed opioid as the early group, and the remainder as the late group.

**Results:**

Among 103 patients who received naldemedine, 98 fulfilled the eligibility criteria. The median age was 68 years and 48% of the patients were female. Median performance status was 3, and the median oral intake was 50%. The median duration of naldemedine administration and overall survival were 25 and 64 days, respectively. The incidence of diarrhea in the early group (*n* = 26) was significantly lower than in the late group (*n* = 72) (3.9% vs. 22.2%, *p* = 0.02). Daily defecation counts increased after late (median 0.43 to 0.88, *p* < 0.001), but remained stable after early naldemedine administration (median 1.00 to 1.00, *p* = 0.34). Constipation after the diarrhea was resolved was common (53%), especially among patients who stopped naldemedine (78%). The diarrhea was improved within three days in 92% of patients who stopped other laxatives.

**Conclusions:**

The early administration of naldemedine is beneficial because it reduces adverse events including diarrhea. Diarrhea caused by naldemedine can be effectively managed by stopping other laxatives while continuing naldemedine.

## Background

Opioid-induced constipation (OIC) is one of the most common and troublesome adverse events in patients using opioids, occurring in > 50% of patients using opioids if prophylaxis is not provided [1, 2]. Although probabilities differ depending on drugs and the administration route [3, 4], almost all opioid analgesics can cause OIC. Unlike other adverse events such as nausea/vomiting or somnolence, the continued administration of opioids does not result in resistance to OIC [5]. Prolonged constipation can cause appetite loss, nausea and vomiting, abdominal pain, and delirium [6]. In addition to the direct impairment of quality of life (QOL), these adverse events interfere with pain control [7] by disabling oral analgesic intake or discouraging patients from using rescue doses. Thus, OIC management is essential to maintain the QOL of patients with cancer pain controlled by opioids.

Opioids exert analgesic effects mainly by activating opioid receptors in the central nervous system. The parallel activation of mu-opioid receptors located on submucous and myenteric neurons of the intestinal tract suppress normal bowel movement [8]. Such opioid-induced bowel dysfunction (including OIC) mediated by the enteric nervous system starts very soon after starting opioids [9]. The activation of mu-opioid receptors in the enteric nervous system decreases intestinal secretion, sphincter relaxation, and longitudinal coordinated contraction of the digestive tract [8]. The peripherally-acting mu-opioid receptor antagonist (PAMORA) is a large molecule with side chains that confer low ability to pass through the blood-brain barrier and this enables selective inhibition of peripheral opioid receptors. This antagonist normalizes bowel function by inhibiting opioid binding to the enteric nervous system, without diminishing the analgesic effect of the opioids [9]. Currently available PAMORA such as methylnaltrexone [10], alvimopan [11], naloxegol [12], and naldemedine [13] can effectively treat OIC. Oral naloxone is also similarly useful with its high first-pass effect [14].

Naldemedine, the first approved PAMORA in Japan, non-competitively inhibits mu-, delta-, and kappa-opioid receptors [15]. A phase 3 randomized placebo-controlled trial of naldemedine for cancer patients with OIC significantly increased spontaneous bowel movements [13]. Major toxicities include diarrhea (19.6%), malaise (4.1%), vomiting and decreased appetite (3.1% each), and 9.3% of the participants had to discontinue the drug due to adverse events [13]. The incidence of diarrhea dose-dependently increases and the diarrhea can be severe enough to result in discontinuation of the drug [16]. Other industry-sponsored clinical trials have shown that naldemedine is similarly effective and safe [13, 17]. However, these trials included participants who were carefully selected, and data of predictive factors or detailed clinical course of adverse events were lacking. Thus many clinical questions remained, such as the appropriate strategies to manage adverse events such as diarrhea, the clinical course after diarrhea is resolved, and risk factors for adverse events. Therefore, we investigated real-world evidence of naldemedine effects, to identify strategies to prevent and appropriately manage adverse events including diarrhea, which is the most frequent adverse event associated with naldemedine.

## Methods

This multi-center, retrospective cohort study comprised reviews of electronic charts performed in April 2018. The protocol included a waiver of the need for written informed consent, which was approved by the institutional review boards at all participating centers.

### Study participants

We included patients with cancer who received palliative care at Teikyo University Hospital (Tokyo, Japan) or Toda-chuo General Hospital (Saitama, Japan), received regular opioids, and had taken at least one dose of naldemedine between June 2017 and March 2018. Study period was determined to enroll approximately 100 participants from when naldemedine became available in the participating centers. Initiation and termination of naldemedine were decided by each attending physician. Patients on naldemedine before initially presenting at the participating centers or who (or whose family) declined to participate were excluded from the study.

### Assessments

We evaluated baseline characteristics including age, sex, Eastern Cooperative Oncology Group performance status (ECOG-PS), primary cancer site, organs with cancer involvement, dietary intake, body mass index (BMI), concomitant laxatives, prescribed opioids and doses, and reasons for opioid prescriptions as potential predictors of adverse events.

We also assessed administration of naldemedine and other laxatives, daily defecation counts on days − 7 through + 7 since starting naldemedine, average daily defecation counts before/after starting naldemedine for each patient, the incidence and severity of adverse events within seven days defined by the common terminology criteria for adverse events (CTCAE) version 4.0, reasons for naldemedine discontinuation, and overall survival. In this study, diarrhea was defined as bowel movements with the Bristol stool scale type 6 or 7, aligned with the definition in the CTCAE version 4.0 (“a disorder characterized by frequent and watery bowel movements”). These variables had been recorded by each attending physician or medial staff, as a part of routine assessment.

The primary endpoint of this study was the incidence of diarrhea according to the baseline characteristics. Secondary endpoints included the duration of naldemedine administration, daily defecation counts before and after starting naldemedine, duration and severity of diarrhea as an adverse event of naldemedine, other adverse events, and the incidence of constipation (defined as absence of defecation for three consecutive days) within 7 days after recovery from diarrhea. The participants were assigned to either a group that started naldemedine within three days (early) or more than three days (late) after the first opioid dose. This threshold was based on the finding that bowel dysfunction can become evident as early as three days after opioid initiation [9].

### Statistical methods

The primary endpoint was analyzed using the chi-square test (if the expected value of any cell was < 5, Fisher’s exact test was applied instead), followed by stepwise multivariate analysis. The incidence of diarrhea in the late group was compared with published data to examine potential bias. Daily defecation counts before and after naldemedine administration were compared using the paired t-test (if the data does not follow normal distribution, Wilcoxon signed rank test was applied instead). Overall survival and the duration of naldemedine administration were estimated from Kaplan-Meier curves. Patients who were alive at the time of data acquisition were censored from the overall survival analysis. Patients who continued naldemedine were censored from the analysis of the duration of naldemedine administration. Patients lost to follow-up were also censored at the last visit. Continuous and categorical variables were compared between groups using the t-test (if the data does not follow normal distribution, Wilcoxon signed rank test was applied instead) and Fisher’s exact test, respectively. If any data of daily defecation counts from day − 1 to day + 1 from the first naldemedine dose of a patient was missing, the patient was excluded from analysis. Similarly, patients with insufficient data of specific variables were excluded from corresponding analyses. All tests were two-sided with a significance level of 0.05. All data were analyzed using JMP Pro version 12.0 software (SAS Institute, Cary, NC).

This study was designed to identify a predictor of 20% prevalence and a relative risk > 4, assuming that the overall incidence of diarrhea would be 20% based on previous findings [13]. Ninety patients were required to detect a difference in the incidence of diarrhea between the groups with 80% power for a significance value of 0.05. Assuming that some data would be missing in 10% of patients, a sample size of 100 was determined.

## Results

### Patient characteristics

**Fig. 1:**
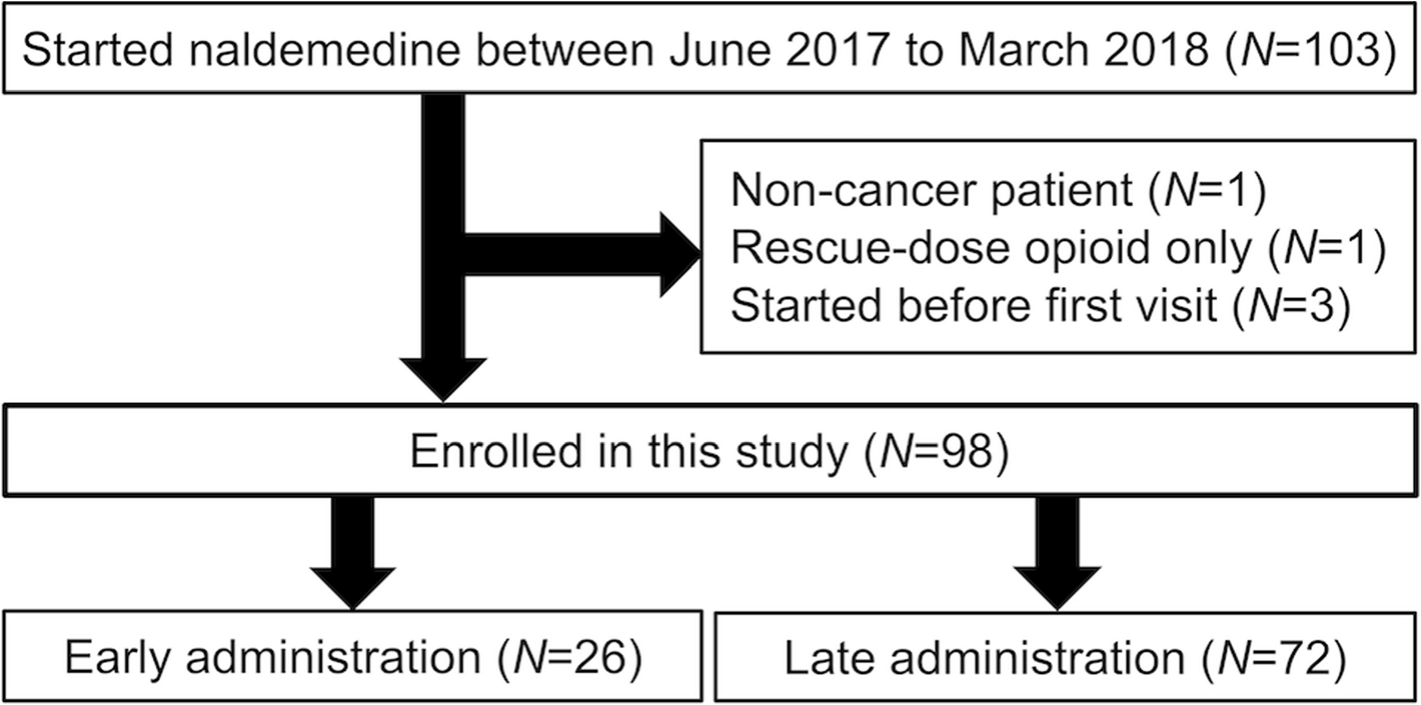
Flowchart of the study

Among 103 patients who received naldemedine at the participating centers, 98 fulfilled the eligibility criteria of this study (Fig. 1). All the participants were Japanese. The median age was 68 years and the number of females was 47 (48%). Primary sites of the cancers were diverse. The median number of organs with distant metastasis was 2, and 26 (27%) and 12 (12%) patients had peritoneal and brain metastasis or involvement, respectively. Performance status of 50 (52%) of the patients was 3–4, and the median oral intake was 50%. Prescribed opioids were morphine or oxycodone in 73% of patients. The median oral morphine equivalent daily dose (MEDD) was 30 (range, 5–480) mg, and the median elapsed time from starting opioids to the first dose of naldemedine was 23 (range, 0–1218) days (Table 1 and Additional file 1).

**Table 1:** Baseline characteristics of study participants

Category	Subcategory	Early group	Late group	Total
Total		26	72	98
Median age (range), y		66 (23–84)	70 (33–87)	68 (23–87)
Sex	Female	10	37	47
	Male	16	35	51
ECOG-PS	1	10	13	23
	2	3	22	25
	3	7	24	31
	4	6	13	19
Primary site of cancer*	Lung	5	16	21
	Colorectum	5	13	18
	Pancreas	3	12	15
	Head & neck	0	6	6
	Uterus	2	3	5
	Ovary	0	5	5
	Other	11	20	31
Median number of distant metastases (range)		2 (0–5)	2 (0–6)	2 (0–6)
Dietary intake^†^	0–10%	3	15	18
	20–40%	8	18	26
	50–70%	5	12	17
	80–100%	10	20	30
BMI^‡^	> 25	6	14	20
	18.5–25	17	37	54
	< 18.5	3	18	21
Prescribed opioid	Morphine	4	9	13
	Oxycodone	16	43	59
	Fentanyl	4	14	18
	Other^§^	2	6	8
Median oral morphine equivalent dose (range), mg/day		19.5 (10–72)	42.5 (5–480)	30 (5–480)
Target symptom of opioid	Pain	23	67	90
	Dyspnea	3	5	8
Median number of laxatives (range)		0 (0–2)	1 (0–3)	1 (0–3)

*BMI* body mass index; *ECOG-PS* Eastern Cooperative Oncology Group performance status

*Three patients in the late group had two types of cancer. ^†^Dietary intake was not assessed in seven patients of the late group. ^‡^BMI was not assessed in three patients of the late group. ^§^Other opioids: tramadol (n = 3 in the late group), hydromorphone (n = 2 in the early group), codeine (n = 2 in the late group), and methadone (n = 1 in the late group)

Twenty-six and 72 patients started naldemedine within three days (early) and more than three days (late) after opioid initiation (Fig. 1). The median MEDD of the early and late groups were 19.5 and 42.5 mg, respectively (*p* = 0.005). The late group started naldemedine at a median of 52 (range, 4–1218) days after starting opioids. In the early group, 27% of the patients took any laxatives at baseline, whereas 63% of the late group used baseline laxatives. The median number of concomitant laxatives at baseline were 0 in the early group, and 1 in the late group (*p* = 0.01). Other baseline characteristics did not significantly differ between the groups.

### Administration and adverse events of naldemedine

At data cutoff of April 20, 2018, the median follow-up time for alive patients was 60 (9–372) days. The median duration of naldemedine administration and overall survival were 25 (range, 1–260) and 64 (range, 2–372) days, respectively. Naldemedine-related adverse events developed in 20 (20%) patients, including diarrhea (*n* = 17), abdominal pain (*n* = 4), nausea/vomiting (*n* = 3), and AST/ALT elevation (n = 1). Naldemedine was stopped in 73 (74%) patients. Most patients continued naldemedine until they could no longer take oral drugs. The reasons for stopping naldemedine comprised oral intake inability (77%), adverse events (11%), termination of opioids (7%), transfer to another hospital (2%), and unknown (2%). Adverse events that led to naldemedine discontinuation were diarrhea in five patients (diarrhea in two patients was caused by factors other than naldemedine) and abdominal pain, nausea, and AST/ALT elevation in one patient each.

The median duration of naldemedine administration and overall survival were 38 and 71 days, respectively, in the early group, and 24 and 59 days, respectively, in the late group. Overall survival and the duration of naldemedine administration did not significantly differ between the groups. Adverse events related to naldemedine occurred more frequently in the late, than the early group (3.9% vs. 26.4%, *p* = 0.01). All eight of the patients who discontinued naldemedine due to adverse events were in the late group.

### Incidence and severity of diarrhea

Diarrhea occurred within seven days from starting naldemedine in 17 (17%) patients, and the median duration of diarrhea was 2 (range, 1–5) days. The characteristics of the 17 patients with diarrhea were as follows: median age, 67 years; female, 8/17 (47%); poor (3–4) ECOG-PS, 11/17 (65%); median oral intake, 25%; morphine or oxycodone, 12/17 (71%); median MEDD, 48 mg.

**Fig. 2:**
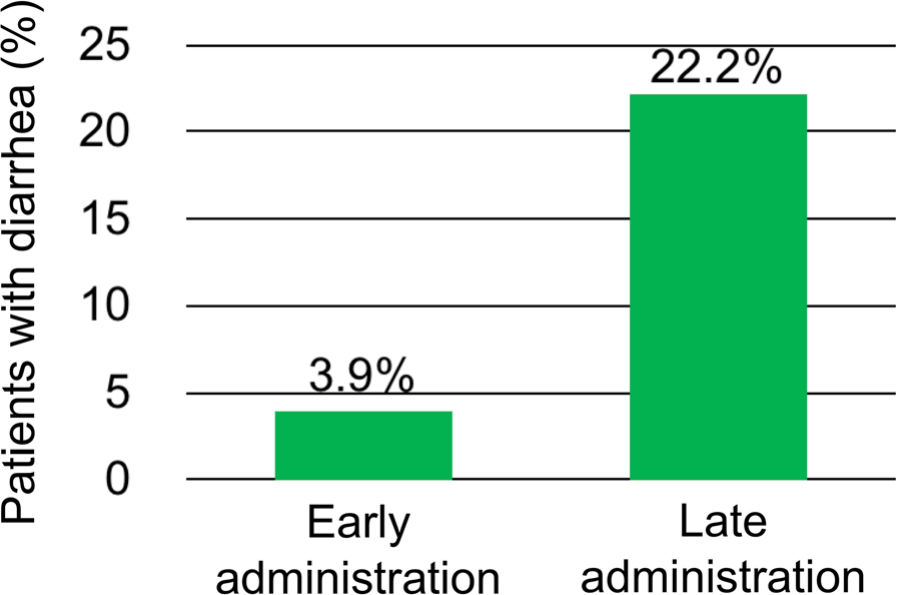
Incidence of diarrhea by group

In the early group, diarrhea occurred in only one patient, whereas sixteen patients experienced diarrhea in the late group. The incidence of diarrhea was significantly lower in the early, than in the late group (3.9% vs. 22.2%, *p* = 0.02; Fig. 2). The severity of the diarrhea was Grade 1 in the patient of the early group, and Grade 1, 2, and 3 in nine, four, and three patients, respectively, in the late group. Other variables in multivariate analyses including age, sex, ECOG-PS, prescribed opioids and doses, baseline dietary intake, and baseline defecation counts did not predict diarrhea.

### Daily defecation counts

**Fig. 3:**
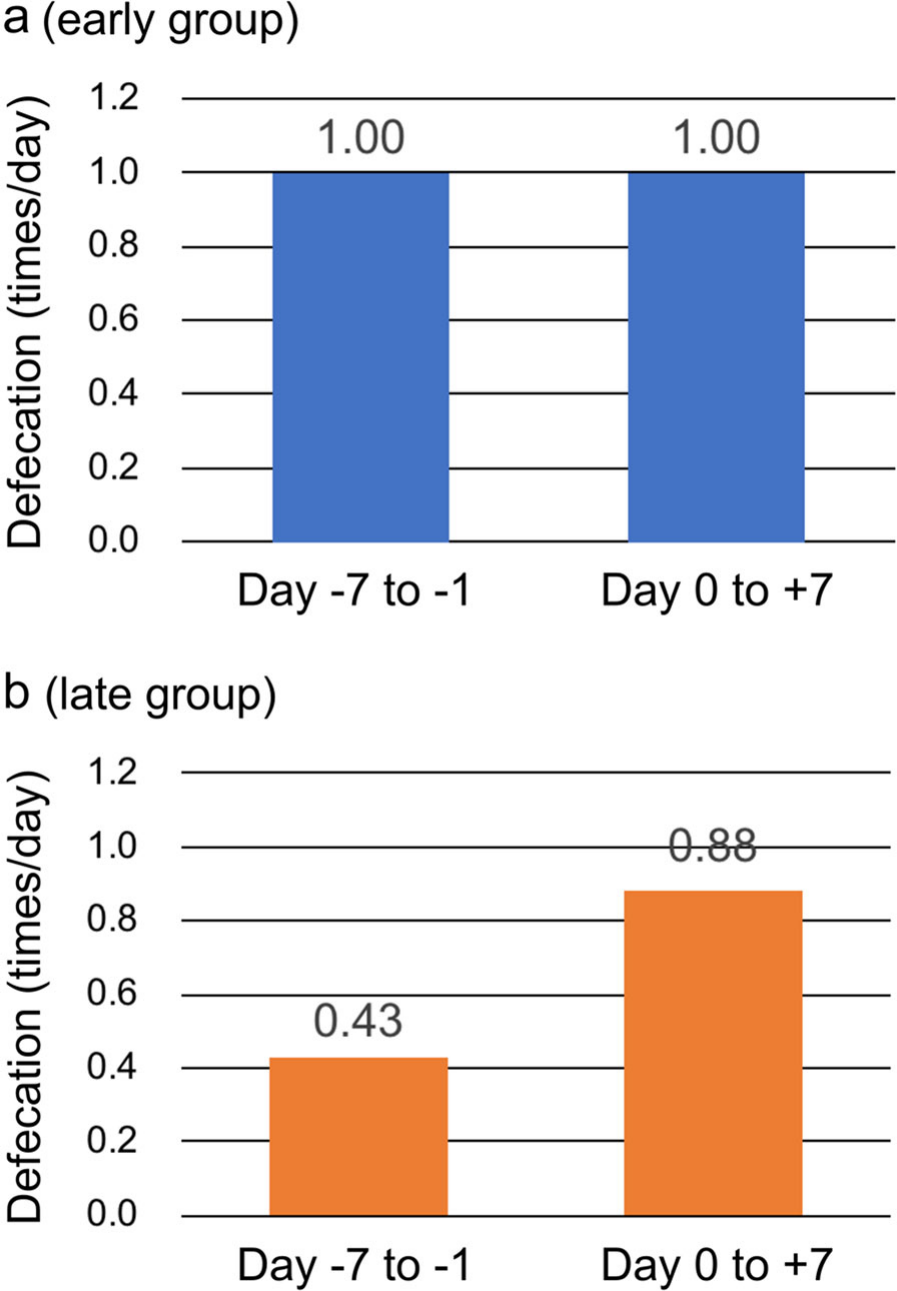
Median number of daily defecations before and after first dose of naldemedine. Early (**a**) and late (**b**) groups

We excluded 19 patients (four and 15 in the early and late groups, respectively) with insufficient information about daily defecation counts. Among the 79 patients analyzed, the baseline defecation counts of 27/79 (34%) were < 0.4 times/day. Of the 27 patients with daily defecation counts < 0.4, the median age was 70 years, 15/27 (56%) were female, 16/27 (59%) had poor (3–4) ECOG-PS, median oral intake was 60%, morphine or oxycodone was prescribed for 23/27 (85%) patients, and the median MEDD was 30 mg. Other baseline characteristics and baseline defecation counts did not significantly correlate, but daily defecation counts were significantly lower in the late, than the early group (*p* = 0.03). In the early group, the median daily defecation counts were 1.00 (average 1.24; standard deviation [SD] 1.54) before naldemedine administration and 1.00 (average 0.95; SD 0.67) after starting naldemedine (Fig. 3a). In the late group, the median daily defecation counts were 0.43 (average 0.60; SD 0.67) before naldemedine administration and 0.88 (average 1.00; SD 0.67) after starting naldemedine (Fig. 3b). Naldemedine significantly increased daily defecation counts in the late group (*p* < 0.001), but not in the early group (*p* = 0.34). Daily defecation counts between groups became similar after the initiation of naldemedine (*p* = 0.13).

### Clinical course after diarrhea

Sixteen of the 17 patients who developed diarrhea also received other laxatives, including magnesium oxide (*n* = 11), lubiprostone (*n* = 7), picosulfate (*n* = 3), senna (*n* = 2), and lactulose (n = 1), and seven patients received more than one laxative. According to the decision of the attending physician, 9/17 (53%) patients discontinued naldemedine and 12/17 (71%) stopped other laxatives. Nine of seventeen (53%) patients subsequently developed constipation after the diarrhea was resolved. Subsequent constipation was quite frequent (83%) in six patients who stopped naldemedine and other laxatives, and two of three (67%) patients who stopped naldemedine developed constipation. Among the patients who continued naldemedine, constipation subsequently occurred in two of six (33%) patients who stopped other laxatives, and in none of two patients who continued with them (Table 2). The duration of diarrhea did not differ regardless of whether naldemedine and/or other laxatives were stopped, although more drugs tended to be stopped for patients with higher CTCAE Grades of diarrhea.

**Table 2:** Clinical course of diarrhea

Stopped naldemedine	Stopped other laxatives	*N*	Duration of diarrhea (days)	Constipation after diarrhea improved
Yes	Yes	6	1, 1, 2, 2, 3, 4	5 (83%)
Yes	No	3	1, 5, 5	2 (67%)
No	Yes	6	1, 1, 1, 1, 3, 3	2 (33%)
No	No	2	1, 3	0 (0%)

## Discussion

The present real-world cohort study showed a significant reduction in the incidence of diarrhea in patients who started naldemedine within three days of starting opioids. Although diarrhea is one of the most common adverse events of PAMORA including naldemedine, effective prophylaxis has not been established. Almost all studies of PAMORA included patients who had already been taken opioids for various periods [10–14, 16, 17]. Diarrhea in such patients is associated with peripheral opioid withdrawal at the intestinal tract induced by PAMORA, and more frequent use of concomitant laxatives for treating preceding OIC. The incidence of diarrhea was 22.2% among the late group, similar to a phase III trial of patients pretreated with regular opioids [13]. The present findings of a low incidence of diarrhea (3.9%) among patients who started naldemedine soon after starting opioids support the hypothesis that PAMORA induction before the formation of peripheral dependence on opioids can prevent diarrhea, which reflects peripheral withdrawal from opioids.

Daily defecation counts were significantly increased by starting naldemedine late. This indicates that naldemedine is consistently effective for OIC in the real-world setting, as well as in clinical trials of select participants [13, 16, 17]. Naldemedine tolerability among patients on regular opioids was also confirmed by the low incidence of discontinuation due to potential adverse events, even though > 20% of the patients developed diarrhea. Further tolerability in patients in the early group was not limited to the incidence of diarrhea as the overall incidence of adverse events was low, and none of these patients discontinued naldemedine due to adverse events. We investigated potential confounders using exploratory univariate analysis including baseline opioid doses in addition to multivariate analyses, because the baseline dose of opioids was significantly higher in the late group. The results of the analyses confirmed that the timing of naldemedine administration impacted the incidence of diarrhea, whereas the baseline dose of opioids did not. Other factors reflecting vulnerability, such as older age or poor PS did not predict the incidence of diarrhea, although frail patients tend to experience more adverse events in general.

Constipation frequently occurred after diarrhea was resolved by naldemedine. This can be explained by the fact that opioid withdrawal, which is the main cause of diarrhea, is a transient process. This mechanism is also applicable to our findings that the incidence of constipation increased among patients who stopped naldemedine upon developing diarrhea. When other laxatives were stopped, the diarrhea of most patients resolved within a few days, irrespective of whether naldemedine was stopped. Our findings indicate that diarrhea occurring after naldemedine initiation should be managed by stopping other laxatives while naldemedine is continued, especially when the diarrhea is not severe. One exception is when opioids reduce pain by suppressing bowel movements. For example, when a patient with peritoneal metastasis develops abdominal pain and diarrhea after starting naldemedine, stopping it to suppress bowel movements again is a reasonable option.

The prophylactic effect of early naldemedine administration cannot be simply compared with the therapeutic effect of late naldemedine for existing OIC. Defecation counts increased once in most patients who started naldemedine late, then decreased and stabilized by continuing naldemedine. In contrast, a transient increase in bowel movements reflecting peripheral withdrawal from opioids was rare among the patients who started naldemedine early. Long-term management is needed for OIC, thus short-term endpoints such as spontaneous bowel movement responder rates [12, 13, 16] are not necessarily appropriate to evaluate the overall benefits of PAMORA. The fact that the daily defecation counts did not significantly change and that daily defecation counts between groups become similar after the initiation of naldemedine suggest that early naldemedine administration somewhat prevents OIC, although a randomized controlled trial is required for confirmation. Bowel dysfunction in patients with cancer has many causes other than opioids, such as low physical activity due to somnolence induced by opioids or the cancer itself, decreased dietary intake, and some types of anticancer chemotherapies. Other concomitant laxatives or non-pharmacological interventions are needed for patients with cancer who have constipation derived from factors other than opioids that cannot be managed by naldemedine.

This retrospective study based on real-world practice has some limitations. First, selection bias which influences the adverse events of naldemedine may exist. The comparison of baseline characteristics between groups indicated that only opioid dose, daily defecation counts and use of concomitant laxatives were significantly differ, whereas general condition refelected by ECOG-PS and dietary intake was similar. Selection bias lead by these findings can be that patients in the late group were more likely to develop the adverse events of naldemedine due to the high dose of opioids. This potential bias rather reinforce the benefit of early administration of naldemedine, when dose of opioid is relatively low. Second, detailed parameters such as straining during defecations or sensation of incomplete evacuation could not be uniformly obtained, because of the retrospective nature of this study. However, this study aimed to investigate effects and safety of naldemedine in the real world, an approach that enabled finding new treatment strategies that had not been evaluated in pivotal clinical trials. Third, potential harm included a financial burden imposed by administering naldemedine to patients who had not yet developed OIC. However, we believe that the high prevalence of OIC [2], the significant impact of OIC on the QOL of patients [7], the economic burden [18], and the magnitude of improving the safe management of OIC justified the early administration of naldemedine.

PAMORA is the etiologic treatment drug for OIC. Since conventional laxatives cannot control OIC in more than one third of the patient using opioids [2], PAMORA can reduce OIC if used routinely with opioid unless a contraindication exists. From the point of view of safer use of naldemedine, this drug should be started within 3 days from the initiation of regular opioid, according to our findings.

## Conclusions

In conclusion, early naldemedine administration after starting opioids can significantly reduce adverse events including diarrhea. Naldemedine-induced diarrhea should be managed by stopping other laxatives while continuing naldemedine. Taking the mechanism of adverse events of naldemedine and other PAMORA into account, new approaches for the safer management of OIC indicated by this study can be potentially extrapolated to other PAMORA. Further prospective studies are warranted to resolve the clinical questions raised in this study.

## Supplementary information

**Additional file 1:** Baseline characteristics and outcomes of individual study participants.

## Data Availability

All data generated or analysed during this study are included in this published article and its Additional file 1.

## Abbreviations

ALT: Alanine aminotransferase
AST: Aspartate aminotransferase
BMI: Body mass index
CTCAE: Common terminology criteria for adverse events
ECOG-PS: Eastern Cooperative Oncology Group performance status
MEDD: Morphine equivalent daily dose
OIC: Opioid-induced constipation
PAMORA: Peripherally-acting mu-opioid receptor antagonist
QOL: Quality of life
SD: Standard deviation
